# The Causes and Consequences of Topological Stress during DNA Replication

**DOI:** 10.3390/genes7120134

**Published:** 2016-12-21

**Authors:** Andrea Keszthelyi, Nicola E. Minchell, Jonathan Baxter

**Affiliations:** Genome Damage and Stability Centre, Science Park Road, University of Sussex, Falmer, Brighton, East Sussex BN1 9RQ, UK; A.Keszthelyi@sussex.ac.uk (A.K.); N.Minchell@sussex.ac.uk (N.E.M.)

**Keywords:** DNA replication, DNA topology, DNA topoisomerases, transcription, fork rotation, fork reversal

## Abstract

The faithful replication of sister chromatids is essential for genomic integrity in every cell division. The replication machinery must overcome numerous difficulties in every round of replication, including DNA topological stress. Topological stress arises due to the double-stranded helical nature of DNA. When the strands are pulled apart for replication to occur, the intertwining of the double helix must also be resolved or topological stress will arise. This intrinsic problem is exacerbated by specific chromosomal contexts encountered during DNA replication. The convergence of two replicons during termination, the presence of stable protein-DNA complexes and active transcription can all lead to topological stresses being imposed upon DNA replication. Here we describe how replication forks respond to topological stress by replication fork rotation and fork reversal. We also discuss the genomic contexts where topological stress is likely to occur in eukaryotes, focusing on the contribution of transcription. Finally, we describe how topological stress, and the ways forks respond to it, may contribute to genomic instability in cells.

## 1. Introduction

Every time a cell grows and divides it has to faithfully duplicate every base pair of DNA in its genome. During this process the DNA replication machinery faces numerous challenges. Different types of DNA structure, stable protein-DNA complexes, and other DNA metabolic processes, such as transcription, can all inhibit or slow ongoing DNA replication.

Replication inhibition can occur through two different pathways. Helicase unwinding of the template DNA and polymerase action can be separately impeded (see [1,2,3] for reviews on this latter process). Inhibition of helicase unwinding comes in two forms. First, certain contexts can sterically block the helicase from unwinding DNA, e.g. collision with other protein complexes tightly bound to the DNA. Alternatively, unwinding can be inhibited by DNA topological stress [4]. In this review we examine the causes and consequences of DNA topological stress on ongoing DNA replication, focusing primarily on events occurring in the eukaryotic system. In particular, we examine how topological stress resulting from transcription influences DNA replication.

## 2. DNA Topological Stress and Topoisomerase Action

DNA topological stress arises due to the intertwining of the two anti-parallel, complementary strands. Under physiological conditions (B form) the two strands are intertwined around each other every 10.4 base pairs [5]. Opening up the DNA without removing the intertwines between the two strands (as occurs during transcription or DNA replication) introduces topological stress into the DNA. On short, naked, linear DNA this stress can diffuse away and off the end of the DNA by the axial spinning of the double stranded DNA. However, in eukaryotic cells this motion can be hindered by the rotational drag generated by protein-DNA complexes. These structures could include RNA polymerase and associated RNA processing factors, stable protein-DNA complexes, different arrangements of the nucleosomal fibre, or proteins that directly link the DNA to relatively immobile structures, such as the nuclear membrane. Such barriers cause accumulation of topological stress along the chromosome. Positive supercoiling stress will impede further unwinding of the strands. Negative supercoiling stress will destabilize B form DNA and promote unwinding. In both cases, the topological stress between the parental strands can be directly removed by the action of topoisomerases. Cells utilize topoisomerase enzymes that relax topological stress by introducing temporary strand breakage into the DNA. Type I topoisomerase nicks one strand, while type II topoisomerases break both strands while passing another section of DNA through the break. Both types of activity allow changes in the extent of linkage between the two strands, before re-ligation, ensuring integrity of the DNA [6,7].

## 3. Topological Stress in the Context of DNA Replication

Resolving topological stress is essential for DNA replication. As the replicative helicase progresses along the DNA it forces the two strands apart. However, its action does not remove the intertwining between the two strands. Therefore, intertwines between the parental strands build up ahead of the replisome, resulting in overwinding and potential distortion of the parental template due to build-up of positive helical stress [8,9]. High levels of such DNA topological stress will impede unwinding of the strands and arrest helicase progression [4,10,11,12]. In eukaryotes the type IB topoisomerase topo I (Top1) and the type II topoisomerase topo II (Top2) are generally utilized to resolve topological stress accumulated during DNA replication.

**Figure 1:**
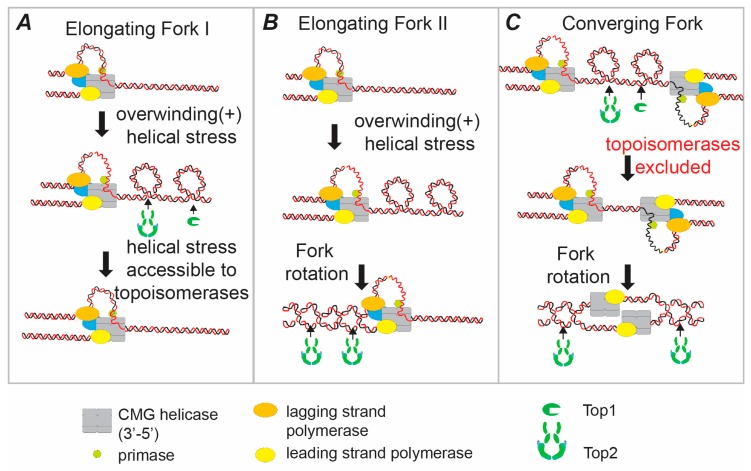
Resolving the topological stress generated by replicative helicase action. During elongation of DNA replication, unwinding of the parental template by the replicative helicase (CMG) separates the parental strands, but does not resolve the intertwining that exists between them. The intertwines between the strands are displaced into the region ahead of the fork leading to overwinding and positive (+) helical stress (shown in figure as positive supercoils). There are two pathways to relax this stress before it arrests ongoing DNA replication—topoisomerase action and fork rotation. (**A**) The tension is directly resolved by the action of either a type IB topoisomerase (such as eukaryotic topoisomerase I (Top1)) or a type II topoisomerase (such as eukaryotic topoisomerase II (Top2)). These topoisomerases act effectively as “swivelases” ahead of the fork. (**B**) Champoux and Been (see text) proposed a second mode of unwinding where the helical tension is relaxed by rotation of the fork to generate catenated DNA sister-chromatid intertwines behind the fork. Although these intertwines should not arrest forward elongation of replication, it is essential that the decatenating type II topoisomerases resolve all DNA catenation before the completion of cell division. (**C**) At the termination of DNA replication when two forks converge, topoisomerases become sterically excluded from the unreplicated DNA. In this case the final few turns of DNA have to be unwound by rotation of the fork relative to the DNA. In eukaryotes the CMG helicase remains bound until they reach the replicated DNA on the other side of the termination zone. Therefore, the two CMG helicase complexes and the leading strand polymerase bypass one another during termination.

Topoisomerase action ahead of the fork, by either type I or type II topoisomerases, relaxes the positive helical stress in the parental strands, preventing topological stress from arresting the progression of DNA replication (Figure 1A). Indeed, topoisomerase activity is essential for progressive DNA replication both in vitro and in vivo [11,13,14,15]. In this model, topoisomerases match the classic concept of a replication “swivelase” [11,13]. However, there are situations where topoisomerases will have potentially limited access to the parental DNA ahead of the fork, most notably as replication forks converge at the termination of DNA replication (see below). If topoisomerase action ahead of the fork were the only pathway to relax topological stress, then DNA replication would be arrested in regions where topoisomerases are inhibited from accessing the template. However, there is a second pathway to prevent topological stress building up ahead of the fork; fork rotation and the generation of a double-stranded intertwine (DNA catenane) behind the fork (Figure 1B) [16].

## 4. Fork Rotation and the Generation of Double-Strand Intertwines—DNA Catenanes

The rotation of the replication fork relative to the unreplicated DNA transfers the intertwines between two single strands from ahead of the fork to the region behind the fork by generating intertwines between the double strands of the newly-synthesized DNA (Figure 1B). Therefore, fork rotation allows elongation to occur without topoisomerase action ahead of the fork. However, this action means that the replicated sister-chromatids become intertwined. Such intertwinings, known as pre-catenanes, mature to full DNA catenanes following the completion of DNA replication. The DNA catenanes generated by fork rotation must be resolved by a type II topoisomerase before the replicated chromosomes can be separated during cell division [4].

Thus, there are two pathways to prevent topological stress building up ahead of an elongating fork and arresting DNA replication. This leads to the question of how frequently the two pathways are utilized to relax topological stress and prevent the arrest of replication elongation? Key experimental insights into when fork rotation is utilized to relax topological stress have come from studies examining the termination of DNA replication.

## 5. Termination of DNA Replication

At the termination of DNA replication two replisome holoenzymes converge to complete the replication of adjacent replicons. During this process the complexes must overcome the topological stress generated between replisomes to fully unwind the DNA. In this situation topoisomerases are likely to be sterically excluded from the unreplicated DNA by the presence of the two converging replisomes (Figure 1C). Together, this leads to high, induced levels of DNA topological stress that could potentially stall replication in its final stages. During this process, every nucleotide needs to be accessible to polymerization. Therefore, the terminating replisomes must be prevented from arresting over any unreplicated DNA. Both classical and recent studies of eukaryotic termination of DNA replication have shown that fork rotation is central to completing replication. Classical studies of Simian virus 40 (SV40) replication have indicated that the replication machinery utilizes fork rotation to unwind the final 100–150 base pairs [17,18]. This allows the induced topological stress to be overcome and the template to be completely unwound and replicated. The final replication intermediates of this process are the intertwined SV40 circular chromosomes generated by fork rotation. These catenated molecules are then decatenated by topoisomerase II to generate the fully-replicated daughter chromosomes. Recent analysis of fork convergence on episomal plasmids in *Xenopus* extracts has also significantly extended our understanding of replication termination [19]. This study demonstrated that during termination the two rotating replisomes do not dissociate from the DNA when the replisomes come together. Rather, the terminating replisomes, travelling 3′-5′, slide past one another, stopping only when they appear to contact the replicated DNA of the opposing strand (Figure 1C) [19]. In summary, fork rotation is used at termination to overcome the induced topological stress between the complexes, preventing stalling. Here the rotating replisome is not sterically blocked by the converging replisome elongating on the other strand. Rather, the complexes appear to bypass one another.

How often fork rotation is utilized for unwinding outside of termination is still relatively unexplored. Data from in vitro bacterial replication systems indicate that the replication fork rotates relatively freely during elongation [20,21]. However, evidence from eukaryotes indicates that fork rotation is actively limited outside of termination. Increasing the size of yeast plasmid replicons does not increase the average number of fork rotation events during DNA replication [22], arguing that fork rotation is not generally utilized outside of termination.

This limitation of fork rotation by the eukaryotic replisome could be down to two non-exclusive scenarios [4]. First, the in vivo activity of the type IB topoisomerase I and topoisomerase II ahead of the fork is potentially always sufficient during normal elongation to prevent the build-up of levels of topological stress required to trigger fork rotation. Second, the structure of the replisome itself could be generally resistant to rotation. In this case only levels of topological stress that start to significantly slow replication would be sufficient to overcome this resistance and force the fork to rotate. Presumably, this would be the case during termination.

Whichever scenario is correct, recent work has shown that the Timeless/Tipin complex is a core factor in regulating the frequency of fork rotation during DNA replication. Deletion of either of the yeast homologues of Timeless/Tipin, Tof1/Csm3, dramatically increases the frequency of fork rotation during replication [22]. Therefore, these proteins are required to minimize fork rotation during DNA replication (in the context of Figure 1 Tof1/Csm3 promote usage of Figure 1A and inhibit Figure 1B). The mechanism of how Tof1/Csm3 restrict fork rotation is not yet clear. However, previous studies suggest it could be through either of the scenarios suggested above. Tof1 has been reported to directly interact with Top1 [23]. Tof1 in the replisome could actively recruit Top1 to the unreplicated DNA just ahead of the fork. Other work has shown that Timeless/Tipin proteins help co-ordinate the actions of the helicase and leading strand polymerase [24,25,26]. Potentially, the structural rigidity introduced though coordinating these sub-complexes could inhibit rotation of the replisome.

Altogether, the replication machinery responds to topological stress by rotating the fork. This allows topological stress ahead of the fork to be relaxed without the direct action of topoisomerases and without the need to fully arrest replication (assuming there is not also a sterical block to replication present). However, the extent of fork rotation that occurs in vivo is restricted by Tof1/Csm3 activity (at least in budding yeast). This suggests that fork rotation is limited to contexts where it is absolutely required to supplement topoisomerase activity ahead of the fork. In the next section we will review which genomic contexts may require fork rotation to prevent fork arrest due to topological stress.

## 6. Sterical Blocks to Replication Induce Topological Stress and Fork Rotation

**Figure 2:**
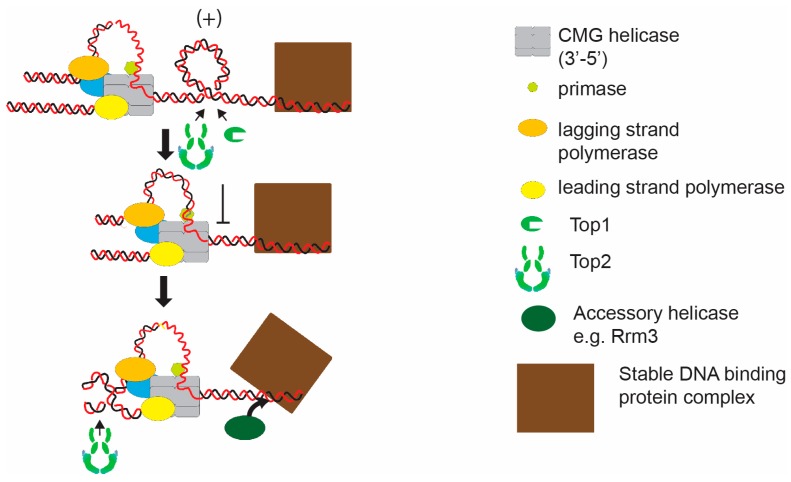
Hypothetical model of induction of topological stress at a stable protein-DNA complex. As the replisome approaches a stable protein-DNA complex, topoisomerases are inhibited from acting between the complex and the converging replisome. Potentially, this will initiate fork rotation to facilitate unwinding. In this case replication fork elongation will also be facilitated by the action of an accessory helicase, such as Rrm3, which will promote displacement of the stable protein-DNA complex.

The situation occurring at the termination of DNA replication provides some predictions of how replisomes could respond when they collide with other protein complexes. At termination, the combination of a local build-up of topological stress and the inhibition of topoisomerase access causes the fork to rotate as DNA unwinding becomes inhibited. Potentially, the same context arises when replication forks converge on stable protein-DNA structures (Figure 2).

The stable DNA binding of protein complexes such as inactive origins and kinetochores is known to sterically block elongation of the fork [27]. Replication through such complexes utilizes specialist displacement helicases, such as the Pif1 family helicase Rrm3, to displace the structures and allow rapid elongation through the site. However, the stability of protein-DNA binding could also lead to topological stress as the replication fork converges. The DNA bound complex could provide both a barrier to diffusion of topological stress and also occlude access of topoisomerases to the region between the replisome and the protein complex (Figure 2). Indeed, the addition of stable protein-DNA structures to episomal plasmids does increase the frequency of fork rotation during DNA replication [22]. Deletion of *rrm3* and stabilization of protein-DNA binding, further increases fork rotation on stable protein-DNA plasmids [22]. This argues that the level of topological stress incurred at the replication fork on passage through stable protein-DNA is frequently sufficient to cause fork rotation (Figure 2). The frequency of fork rotation at these sites is likely related to the binding stability of the protein-DNA complex.

Potentially, the most significant impediments to DNA replication occur due to RNA transcription. RNA transcription complexes could act as both sterical and topological blocks to DNA unwinding by replication. Other recent reviews have extensively discussed how transcription could impede unwinding DNA either through direct collision, or through the generation of non-B form structures and R loops [28,29]. In this review we will focus on how topological stress induced by transcription can disrupt DNA unwinding.

## 7. Transcription and DNA Topological Stress

Transcription unwinds the DNA template to gain access to the coding strand and generate nascent transcripts. The twin supercoiled domain model [30] stipulates that the unwinding of the DNA by the transcription machinery results in positive supercoiling ahead of the transcription bubble. Behind the complex, base-pairing of DNA emerging from the bubble causes negative supercoiling (to compensate for the B-form twist of the DNA). Theoretically these supercoiling domains will only be formed when the RNA polymerase is prevented from rotating relative to the DNA. However, due to the extensive binding of processing factors to the nascent RNA, the RNA polymerase holoenzyme is likely to be, generally, sufficiently rigid to prevent free rotation of the polymerase.

In this model the two domains ahead and behind the transcription bubble only exist while the RNA polymerase is stably bound to the DNA. Without the binding of the large RNA polymerase holo-complex, the positive and negative supercoiling domains will diffuse together and potentially cancel out. With the elongating RNA polymerase stably bound, topoisomerase action is required to relax the topological stresses generated. If allowed to build up the positive helical stress ahead of the RNA polymerase could arrest transcription. Whether accumulation of topological stress occurs at a transcript likely depends on its chromatin environment and position. Denser nucleosome packaging would likely impede topological stress diffusion [31]. Recent studies of global genome architecture have shown that chromosome fibres are organized into distinct higher domains, separated by insulator structures [32]. These insulators inhibit heterochromatin spreading and most likely act as a topological barrier to the diffusion of transcription induced topological stress. Direct evidence of where topological stress is prevalent in eukaryotic genomes has been provided by the use of psoralen intercalation as a marker for local underwinding [33,34,35]. These studies have confirmed that transcriptional activity correlates with regions of topological stress and that regions proximal to telomeres appear to be less affected by topological stress (presumably due to helical rotation at the end of chromosomes). Future studies will hopefully be able to define more exactly how chromatin structure regulates the local build-up of topological stress. Apart from global chromosomal architecture and position, other factors can influence local accumulation of helical stress. In budding yeast loss of both Top1 and Top2 activity causes a rapid cessation of transcription in the highly expressed rRNA genes but only modest changes at shorter tRNA genes or lower expressed RNA pol II genes. This indicates that topoisomerase activity is primarily required to relax topological stress at highly-expressed genes [11]. At other types of genes presumably topological stress does not build up sufficiently to arrest transcription. More recently, genome wide analysis has shown that loss of Top2 preferentially inhibits transcription at long genes [36]. This suggests that longer transcripts generate higher topological stress, leading to increased dependence on topoisomerase action.

Thus, like DNA replication, transcription introduces topological stresses into the DNA template. Collisions between the two processes can lead to distinct consequences for replication-mediated DNA unwinding. These are dependent on their relative directions of travel; i.e. the same direction (co-directional collisions) or converging, opposing directions (head-on collisions). Co-directional replication-transcription collisions can impact on DNA unwinding at the replication fork in several ways. A co-directional collision could lead to sterical obstruction of replisome progression, particularly if the RNA polymerase progression is paused [37]. Paused RNA polymerase could, potentially, have the same consequences for DNA replication as the stable protein-DNA structures discussed above. In addition, the negative supercoiling generated behind the transcription bubble promotes generation of non-B DNA structures. Structures, such as G4 DNA and R loops, are likely to present distinct challenges for DNA unwinding [38]. Accessory helicases are likely required to unwind these structures before DNA replication can progress [39,40]. However, these unwinding problems are primarily caused by changes in DNA base pairing rather than being due to topological stress.

**Figure 3:**
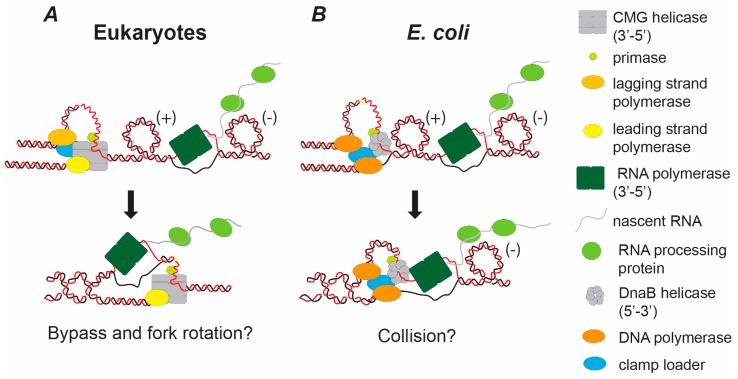
Hypothetical models of the consequences of topological stress and head-on replication-transcription conflicts in eukaryotes and *E. coli*. Head-on collisions between the DNA replication machinery and active transcription will lead to high levels of positive (+) super-helical stress ahead of the fork. There will also be negative (−) superhelical stress behind the transcription bubble. Analogous to the termination of DNA replication, the converging replication and transcription machineries will progressively sterically occlude topoisomerases from acting on the positive (+) super-helical stress between the complexes, inhibiting unwinding. This is likely to initiate replication fork rotation. As the replication and transcription machineries fully converge there are likely to be distinct consequences in eukaryotes (**A**) compared to *E. coli* (**B**). (**A**) In eukaryotes both the CMG helicase and the RNA polymerase translocates 3′-5′. These will converge on different strands. If the complexes act the same way as converging complexes in termination they will bypass one another. (**B**) In contrast, in *E. coli* the replication helicase DnaB translocates 5’-3’, travelling on the same strand as the converging RNA polymerase travelling 3′-5′. Therefore, these complexes will collide unless the RNA polymerase is displaced.

Transcription-related overwinding problems primarily occur only during head-on transcription-replication collisions. The head-on collision between the replication and transcription machineries is likely to cause both sterical and topological problems for DNA unwinding. Both replication and transcription generate overwinding, positive helical stress, ahead of the elongating complexes. The head-on collision resembles the situation occurring between converging replication complexes during termination; a local build-up of topological stress between the converging complexes with increasing inhibition of topoisomerase action (Figure 3 compared to Figure 1C). The induced overwinding could arrest DNA unwinding before the two complexes collide. However, studies in bacteria show that converging replication and transcription machineries do physically come together [41,42]. Therefore, like termination, induced topological stress between the converging complexes does not prevent their collision. During termination this is facilitated by fork rotation. Does fork rotation also occur to overcome the topological stress induced by a head-on replication-transcription collision? 

When considering this point, it is important to note that converging replication-transcription conflicts are potentially distinct events in bacteria and eukaryotes. In *Escherichia coli* the replicative helicase DnaB is a 5’-3’ helicase associated with the lagging strand [43]. In eukaryotes the CMG replicative helicase travels 3′-5′ and is, therefore, associated with the leading strand. Since RNA polymerase primarily translocates the 3′-5′ direction on the transcribed strand, it will presumably act as a lagging strand block to replication [44]. Therefore, in *E. coli*, DnaB and the converging RNA polymerase will come together on the same strand of DNA (Figure 3B) and physically collide. This argues that some form of displacement mechanism will be important for overcoming such conflicts. In contrast, the eukaryotic CMG helicase and RNA polymerase are translocating on different strands. This potentially allows the CMG driven replisome and the RNA polymerase to simply bypass one another (analogous to termination) (Figure 3A). Since DNA unwinding during termination is facilitated by fork rotation (Figure 1C), it seems likely that this will also occur when the eukaryotic replisome and RNA polymerase holo-enzymes come together.

Interestingly, in eukaryotes there is evidence for frequent fork rotation when DNA replication occurs through sites of transcription [45]. Unlike in plasmids, the double stranded DNA intertwines generated by fork rotation cannot be directly detected on endogenous chromosomes. However, recent chromatin immunoprecipitation (ChIP) analysis of the SMC5/6 complex has shown that it accumulates in specific locations following depletion of Top2 [45]. Since enrichment sites could be removed by re-expression of Top2, these findings argue that SMC5/6 enrichment is an in vivo marker of double stranded intertwining. SMC5/6 was found to be significantly enriched at sites of converging genes. This suggests that intertwines are preferentially generated at these sites due to the high probability of head-on DNA replication-transcription collisions.

## 8. Consequences of Topological Stress on Replication—Fork Reversal versus Fork Rotation

The ability of fork rotation to diffuse topological stress from the unreplicated region ahead of the fork into the replicated region behind the fork has the potential to prevent topological stress from arresting replication progression. However, numerous studies have shown that, in certain contexts, fork reversal of arrested forks occurs in response to induced topological stress.

Fork reversal was originally proposed as a pathway for post replication-repair. In this pathway the displacement and annealing of nascent strands and re-annealing of the parental strands could allow the bypass of a lesion on the leading strand of a replication fork [46]. Extensive cross-linking of replicating DNA with psoralen subsequently enabled the four-way replication structures formed by reversal to be observed directly in vivo in budding yeast by electron microscopy. However, their formation initially appeared to be dependent on the destabilization of the replisome following fork arrest [47]. This suggests that a stable replisome structure prevents fork reversal. Other studies have indicated that certain DNA damage responsive DNA helicases or translocases can act at forks arrested due to DNA damage, and drive the reversal of these forks to facilitate damage bypass (for recent review see [48]). Some of these helicases also genetically support replication past sterical barriers [49]. In some cases, reversal could be regulated by the DNA damage checkpoint [50]. However, other studies suggested checkpoint-independent fork reversal [51,52]. Altogether, fork reversal could be playing an active role in overcoming barriers to replication.

With regards to fork reversal occurring as a response to topological stress, introducing overwinding stress (using a DNA intercalating agent) between two paused replisomes in vitro has been shown to cause fork reversal [10,53]. In budding yeast, in vivo replication structures consistent with reversed forks were observed to occur due to the “gene gating” of transcription [54]. In this context connection of a transcribing gene to nuclear pores was postulated to prevent topological stress diffusion and therefore induce high levels of local topological stress when the replication fork converged on the gene. Here, again, fork reversal was only observed following destabilization of the replisome.

**Figure 4:**
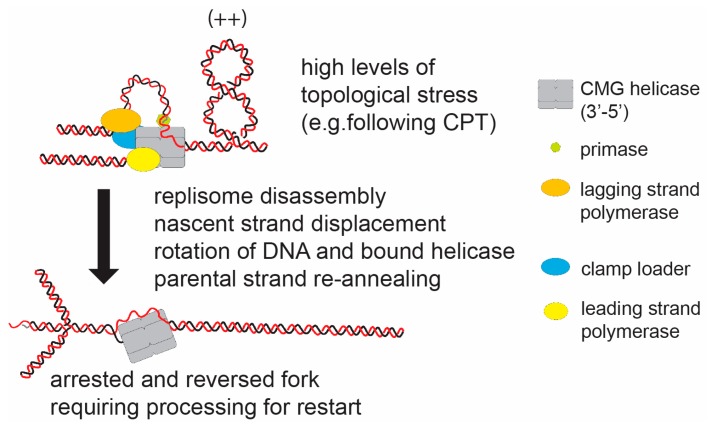
The hypothetical model of fork reversal in eukaryotes in response to DNA topological stress. Replication forks have been observed to reverse in response to high levels of topological stress (e.g., induced by camptothecin (CPT)). In this case the paused replisome must; (i) partially dissemble to expose the nascent strand, (ii) displace the nascent strands (potentially actively); and (iii) promote parental strand re-annealing. The reforming of the intertwined parental strand will relax the positive helical stress ahead of the fork by rotation of the strands and bound CMG helicase. Potentially, this is a stable structure, which will require active structuring to re-form an active fork structure.

However, as the number of contexts screened for reversed forks by psoralen cross-linking and electron microscopy has increased, reversed forks have been found to occur in situations where the replication fork is thought to be fully stable [55]. In some of these contexts fork reversal is linked to the incidence of topological stress generated following addition of the topoisomerase 1 poison— camptothecin (CPT) [51]. Interestingly, the extent of overwinding topological stress induced on plasmids by CPT is significantly elevated relative to that generated by the loss of topoisomerase IB activity alone [56]. In addition to inactivating topoisomerase I, CPT treatment also leads to trapping of the enzyme on the DNA. Potentially the trapping of the enzyme by CPT inhibits resolution of supercoiling by active topoisomerase II [56]. In budding yeast, *Xenopus* extracts, and human cells, fork reversal was observed after treatment of sub-lethal doses of CPT without the formation of double-strand breaks (DSB) occurring (Figure 4) [51]. Reversed forks appeared particularly enriched at converging (terminating) forks where, presumably, a combination of both CPT and endogenous topological stress would accumulate. Thus, the replication fork appears to have two distinct responses at DNA exhibiting elevated levels of topological stress, fork rotation, and fork reversal.

Therefore, an open question is what factors direct which pathway is taken in response to topological stress? It should be noted that while fork rotation appears to be a universal in vivo response to high levels of topological stress, fork reversal often appears to require that replication is occurring under stressed conditions. This potentially makes the replication fork more prone to full arrest. We speculate that fork rotation is the primary response to levels of topological stress that impede, but do not arrest, ongoing replication. In such contexts fork rotation would allow replication to proceed and allow the bypass of regions where topoisomerases may be inhibited from acting ahead of the fork. Conversely, we would argue that, in contexts where the replication fork is completely arrested by topological stress, then fork reversal may occur. Fork reversal in these contexts has been proposed to stabilize the fork until the impediment to replication has been resolved [49].

## 9. Genome Instability and Topological Stress

Generally, ongoing DNA replication is minimally affected by the increase in topological stress generated by loss of either Top1 or Top2, individually. The redundancy of the action of these two enzymes during DNA replication is, however, clearly illustrated by the rapid cessation of replication when both are inhibited [11,12,57]. However, here we have described contexts where the action of both topoisomerases ahead of the fork would be insufficient to relax induced topological stress, inhibiting the unwinding of the parental DNA during DNA replication. We also discuss how fork rotation could potentially overcome the topological stress at these sites, allowing further elongation and preventing stalling of the forks. Interestingly, these contexts are often sites of increased genome instability in cells. For example, conflicts between the DNA replication and transcription machinery are thought to be a primary cause of chromosome instability [37,58,59,60,61]. In higher eukaryotes, sites that are particularly prone to breakage following induced replication stress are termed common fragile sites [1]. Known fragile sites include highly transcribed genes [62], long genes [59], stable protein DNA complexes [27], and DNA prone to secondary structure [63]. All of these structures potentially impede ongoing DNA replication. The exact mechanism of DNA breakage at these sites is still a matter of debate [64]. Current hypotheses suggest that these regions lead to elevated levels of fork arrest. This could lead to DNA breakage by inappropriate processing of the arrested forks [65]. Alternatively, high frequencies of fork arrest in these regions could lead to unreplicated regions of DNA that are then potentially broken during cell division [66].

Given that several fragile site contexts lead to elevated DNA topological stress (highly transcribed and long genes, stable protein-DNA sites), it could be argued that allowing the fork to easily rotate at these sites would minimize fork arrest and the possibility of a double strand breakage. However, our recent work has linked excessive fork rotation and DNA catenation with the incidence of DNA damage. We have shown that increasing the frequency of fork rotation and formation of DNA catenation by deleting *tof1* appears to increase endogenous levels of DNA damage in cells and activates post-replication repair pathways [22]. DNA damage in this context is not caused by breakage of catenated DNA in mitosis since damage can be detected immediately following the S phase. Rather, our data suggests that pre-catenation causes damage by interfering with processes behind the fork. The role of Tof1 in the replisome is still poorly understood and the linkage between excessive fork rotation and DNA damage following *tof1* deletion could be indirect. Loss of Tof1 could destabilize the replisome in a manner that leads to excessive fork rotation and independently to elevated replisome collapse and DNA damage [67]. However, if the link between DNA damage and excessive fork rotation is direct, these observations suggest that fork rotation has both potential positive and negative effects on genome stability in vivo. On one hand, fork rotation facilitates ongoing replication in situations where topological stress could arrest replication. On the other hand, excessive fork rotation may contribute to a higher frequency of endogenous DNA damage, particularly at putative fragile sites.

## 10. Future Perspectives

In summary, we have discussed the contexts where topological stress can interfere with DNA unwinding by the replication machinery. In particular, we have examined the central role that fork rotation appears to have in coping with topological stress and its positive and negative consequences for genome stability. However, at present, many of the possibilities we have outlined in this review are based on indirect evidence. There have been recent advances in in vitro, single molecule studies and genome-wide assays of eukaryotic DNA replication, which can pinpoint how, when, and where replication-induced DNA damage occurs in cells [68,69,70]. Future studies should be able to provide direct mechanistic evidence for these ideas. Such data, we believe, will be essential to gain a holistic view of how the complex structure of DNA is faithfully replicated through countless rounds of duplication.
